# Supplementary material for: The Ethyl Acetate Extract of Phyllanthus emblica L. Alleviates Diabetic Nephropathy in a Murine Model of Diabetes
Source: Int J Mol Sci. 2024 Jun 18;25(12):6686. doi: 10.3390/ijms25126686 (PMC11204328; doi:10.3390/ijms25126686)
Supplement: Supplementary file 1 [file ijms-25-06686-s001.zip › ijms-3021804-supplementary.pdf]

Supplementary

A

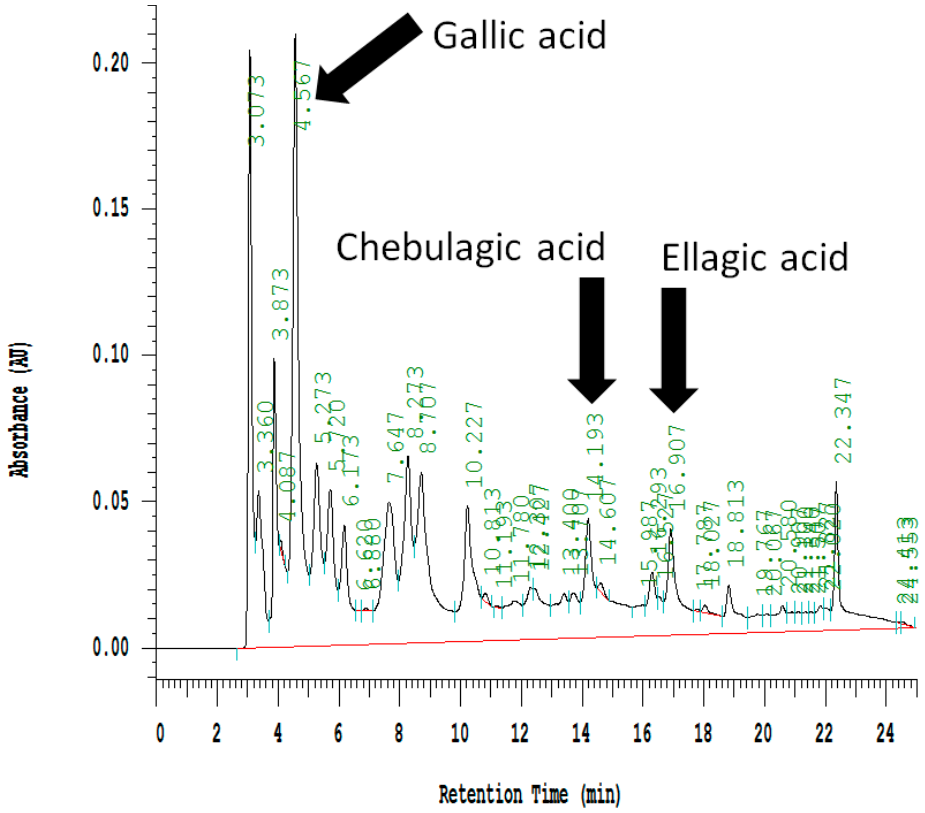

B

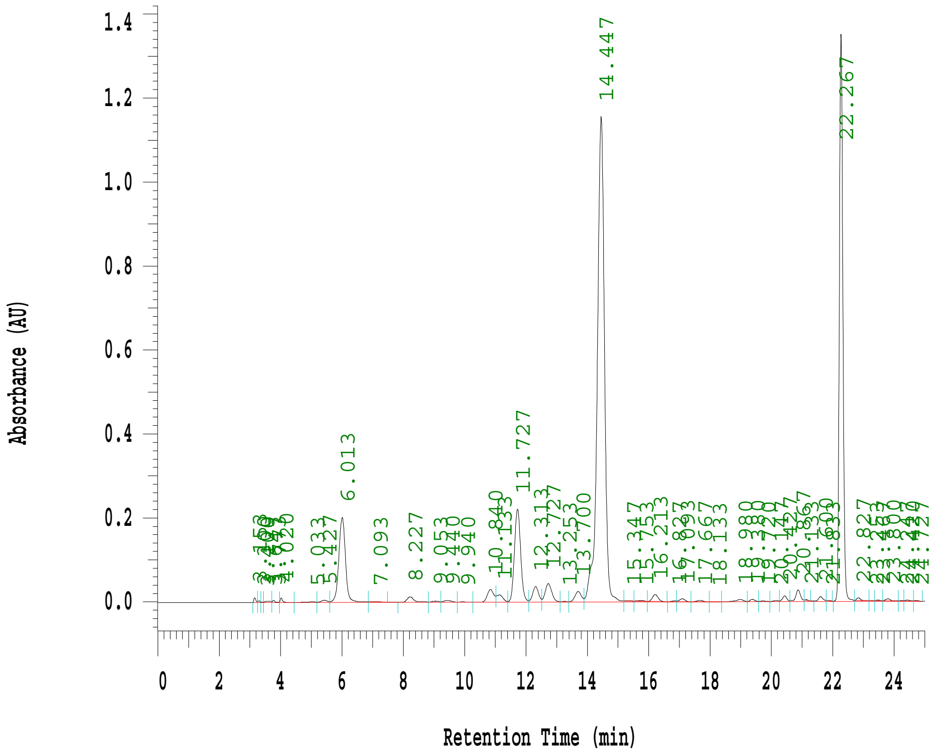

C

Chebulagic acid standard curve & Quantification of EA-4

272 nm						
Standard	87.5	175	350	1050	2100	µg/mL
	513817	849235	2013674	6887050	17526785	
area	414424	800163	1935330	7658348	19353935	
	525380	811422	1968196	8342119	20351540	
AVE	484540.333	820273.3	1972400	7629172	19077420	
RT (min)	14.447					
10.3mg/5mL EA-4	9415066					
	1120.67715	µg/mL				

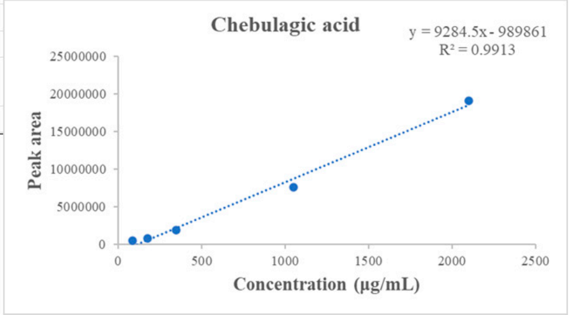

Figure S1. (**A**)~(**C**) High-performance liquid chromatography analysis of (**A**) 2500 ppm ethyl acetate of *Phyllanthus emblica* L. (EPE), (**B**) 10.3 mg/5mL EA-4 of ethyl

acetate of *Phyllanthus emblica* L. **(C)** Chebulagic acid standard curve and Quantification of EA-4.
